# Indoleamine 2,3-Dioxygenase Is Involved in the Inflammation Response of Corneal Epithelial Cells to *Aspergillus fumigatus* Infections

**DOI:** 10.1371/journal.pone.0137423

**Published:** 2015-09-11

**Authors:** Nan Jiang, Guiqiu Zhao, Jing Lin, Liting Hu, Chengye Che, Cui Li, Qian Wang, Qiang Xu, Xudong Peng

**Affiliations:** Department of Ophthalmology, the Affiliated Hospital of Qingdao University, Qingdao, 266003, Shandong Province, China; Hans-Knoell-Institute (HKI), GERMANY

## Abstract

Indoleamine 2,3-dioxygenase (IDO), which is mainly expressed in activated dendritic cells, is known as a regulator of immune responses. However, the role of IDO in immune responses against fungal corneal infection has not been investigated. To evaluate the regulatory mechanisms of IDO in fungal inflammation, we resorted to human corneal epithelial cells (HCECs), known as the first barrier of cornea against pathogenic microorganisms. We found that IDO was significantly up-regulated in corneal epithelium infected with *Aspergillus fumigatus* (*A*. *fumigatus*) and HCECs incubated with spores of *A*. *fumigatus*. Furthermore, IDO inhibitor (1-methyltryptophan, 1-MT) enhanced inflammatory cytokines IL-1β and IL-6 expression which were up-regulated by *A*. *fumigatus* spores infection. Dectin-1, as one of the important C-type lectin receptors, can identify β-glucan, and mediate fungal innate immune responses. In the present study, pre-treatment with curdlan, a Dectin-1 agonist, further enhanced IDO expression compared with *A*. *fumigatus* stimulation. While laminarin, the Dectin-1 specific inhibitor, partially inhibited IDO expression stimulated by *A*. *fumigatus*. Further studies demonstrated inhibition of IDO activity amplified the expressions of inflammatory cytokines IL-1β and IL-6 induced by activation of Dectin-1. These results suggested that IDO was involved in the immune responses of fungal keratitis. The activation of Dectin-1 may contribute to *A*. *fumigatus* spores-induced up-regulation of IDO.

## Introduction

Fungal keratitis (FK) is a serious suppurative ocular disease that can lead to severe vision loss[1]. Lack of suspicion and delayed diagnosis heralds the onset of complications which often lead to permanent loss of vision[2]. In China, one of the main causative organisms in corneal fungal infection is *Aspergillus fumigatus* (*A*. *fumigatus*) and the major risk factor is agricultural trauma. Consequently, the incidence of fungal keratitis increases during harvest season, when there are great chances to expose to airborne soil and contaminated plant materials[3–5].

Immune system plays an important role in recognition and elimination of fungal pathogens through pattern-recognition receptors (PRRs) expressed on innate immune cells in the pathogenesis of fungal keratitis[6]. Dectin-1, as an important member of C-type lectin superfamily, can identify β-glucan, mediate fungal innate immune responses and regulate production of cytokines and chemokines[7]. As the first barrier of cornea against pathogenic microorganisms, corneal epithelial cells play an irreplaceable role in innate immune responses[8]. After being invaded by fungi, PRRs that locate on the corneal epithelial cells, can identify fungi and toxins, initiate immune responses, and recruit neutrophils and macrophages to infection site[7,9]. Infected individuals mount a profound host response to *A*. *fumigatus*, which is characterized by secretion of proinflammatory, chemotactic, and regulatory cytokines, activation and recruitment of neutrophils, macrophages, and T lymphocytes and induction of anti-fungi immunity and clearance[10].

The balance between host and fungal pathogens can influence the prognosis of disease, and excessive inflammation due to not only innate immunity but also adaptive immunity can cause tissue damage and even life-threatening consequences. Ocular immune privilege is necessary for preventing or modifying innate and adaptive immune responses occurring in corneal diseases in order to protect host from excessive damage[11]. Indoleamine 2,3-dioxygenase (IDO), the first enzyme in the kynurenine pathway of tryptophan degradation, plays a key role in inducing protective tolerance against *A*. *fumigatus*[12–14]. IDO is found in many tissues such as cornea, lung, small intestine, placenta and is up-regulated in infected tissues[15–17]. During infection, IDO was firstly described as a mechanism for stopping growth of microorganisms[18]. Thereafter, it was reported that IDO could prevent exacerbation by regulation of T-cell activity in the inflammatory disorder models[19,20]. Expression of IDO in dendritic cells and macrophages can suppress T-cell responses and promote tolerance[21]. In addition to the pivotal role of denritic cells and macrophages, epithelial cells also contribute to the balance between immunopathology and protective immunity to *A*. *fumigatus* via converging on IDO[13]. Taken together, these observations suggested IDO, acting as an immune modulator, may also contribute to the immune response against *A*. *fumigatus* in corneal infections. However, the role of IDO in innate immune response of corneal epithelial cells infected by *A*. *fumigatus* has not been detected. Due to the lack of effective anti-fungal agents, FK has become one of the most urgent infectious keratitis problems. Thus, clarification of the mechanisms underlying the corneal fungal infection processes is important for the treatment and prevention of fungal keratitis. In this study, we investigated the expression and the regulation of IDO in innate immune response of telomerase-immortalized human corneal epithelial cells (HCECs) infected by *A*. *fumigatus* and the possible mechanisms involved in this process.

## Materials and Methods

### Materials and reagents

Dulbecco's modified eagle's medium (DMEM), fetal bovine serum (FBS), 0.25% trypsin, 0.03% ethylenediaminetetraacetic acid solution, and Ham F-12 were purchased from Gibco (San Diego, CA, USA). RNAiso Plus, reverse transcription-polymerase chain reaction (RT-PCR) kits and SYBR Premix Ex Taq™ (Tli RNaseH Plus) were purchased from TaKaRa (Dalian, China). Monoclonal mouse anti-human IDO antibody (Millipore, Billerica, MA) and polyclonal rabbit anti-human Dectin-1 were obtained from Cell Signaling Technology (Boston, MA, USA). 1-methyl tryptophan (1-MT), curdlan and laminarin were obtained from Sigma-Aldrich. Bicinchoninic acid assay and ECL western blotting detection reagents were purchased from Beyotime (Shanghai, China). Phenylmethylsulfonyl fluoride (PMSF) and cell lysis buffer (RIPA) were purchased from Solarbio (Beijing, China).

### Preparation of *A*. *fumigatus*

*A*. *fumigatus* strains (NO 3.0772) were bought from China General Microbiological Culture Collection Center and grown in Sabouraud medium at 28°C for 5–7 days. Spores were harvested from *A*. *fumigatus* cultures as previous described[22,23]. For the experiment of incubation with HCECs, spores were harvested and inactivated for 6 h by treatment with 75% ethanol. After washed 3 times in sterile phosphate-buffered saline (PBS), spores suspensions were adjusted to a concentration of 5 × 10^7^/ml with DMEM/F12. For animal models of keratitis, spores were disrupted with a bacterial L-loop and harvested in 5ml PBS. Pure spores suspensions were obtained by passing the culture suspension through PBS-soaked sterile gauges placed at the tip of a 10ml syringe. Spores were quantified using a hemacytometer and a stock was made at a final concentration of 5 × 10^4^/μl in PBS.

### Cell culture and stimulation

The telomerase-immortalized HCECs were kindly provided by Ocular Surface Laboratory of Xia Men Eye Center (Xia Men, China) and cultured in DMEM/F12 with 10% FBS, 0.075% epidermal growth factor (EGF), 0.075% insulin, and 1% SPA sulfate at 37°C in a humidified atmosphere containing 5% CO_2_. The medium was replaced every 2 days. HCECs suspensions of 1 × 10^5^/ml were seeded into 6-well plates and grew to 70%-80% confluence. HCECs were added with 1 ng/ml 1-methyl tryptophan (1-MT), Dectin-1 ligand (curdlan, 200 μg/ml) and specific inhibitor of Dectin-1 (laminarin, 0.3 mg/ml) for 10 h before inactive *A*. *fumigatus* spores stimulation. The cells in 6-well plates were lysed for extraction of cytoplasmic and nuclear proteins with a nuclear extraction kit (Active Motif, Carlsbad CA), and stored at -80°C for western blot analysis. The cells in 12-well plates were subjected to total RNA extraction for measuring inflammatory cytokine expression by quantitative real-time PCR (qRT-PCR). After various treatments, human corneal cells were harvested and then stained with mouse anti-human IDO antibody (Millipore, Billerica, MA) and analyzed for IDO expression by western blots.

### Examination of human corneal tissue samples

Patients with fungal keratitis at the Department of Ophthalmology (The Affiliated Hospital of Qingdao University) from January 2012 to December 2014 were included. The patients enrolled in the research were clinically confirmed keratomycosis by staining of corneal scrapings, fungal culture (verified *A*. *fumigatus* growth) or confocal microscopy. A total of 31 first-visit patients were divided into three groups according to keratomycosis severity, which were scored visually with aid of a slit lamp: mild group (8 cases, 8 eyes), corneal ulcer diameter ≤ 2 mm, invasion depth of corneal ulcer ≤ 1/3 of the corneal stroma, no hypopyon; moderate group (13 cases, 13 eyes), corneal ulcer diameter from 2 to 4 mm, invasion depth of corneal ulcer from 1/3 to 2/3 of the corneal stroma, no hypopyon; and severe group (10 cases, 10 eyes), corneal ulcer diameter ≥ 4 mm, invasion depth of corneal ulcer ≥ 2/3 of the corneal stroma, with hypopyon. Corneal epithelial scrapings were collected, and the mRNA was analyzed using qRT-PCR. Controls were 6 normal corneal tissues remaining after corneal transplantation. Meanwhile, some healthy corneal tissue samples after eyeball enucleation and samples with fungal infection after corneal transplantation were used for immunofluorescence analysis. These tissue samples were obtained under the patients’ consent and the approval from the Institutional Research Ethics Committee at the Affiliated Hospital of Qingdao University. The protocol for the research conformed to the provisions of the Declaration of Helsinki in 1995 (as revised in Edinburgh 2000).

### Animal models of keratitis and IDO inhibition

All animals were treated in accordance with the Chinese Ministry of Science and Technology Guidelines on the Humane Treatment of Laboratory Animals (vGKFCZ-2006–398) and the Association for Research in Vision and Ophthalmology (ARVO) Statement for the Use of Animals in Ophthalmic and Vision Research. Female C57BL/6 mice (6–8 wk old) were purchased from the Chinese Academy of Medical Sciences (Beijing, China). All corneas were individually inspected under a slit lamp microscope before recruiting into experiments. Only the right corneas were used for model induction and the left eyes were used as untreated controls. The spores were harvested from *A*. *fumigatus* cultures as described above, and adjusted to a final concentration of 5 × 10^4^/μl in PBS. Mice were anaesthetized by intraperitoneal injection of tribromoethanol, and the corneal epithelium was abraded using a 30-gauge needle. A 33-gauge Hamilton syringe was inserted into the abrasion, and 2 μl of 1 × 10^5^ spores in PBS were injected into the corneal stroma as described[24,25]. IDO was inhibited by oral administration of 1-MT (Sigma-Aldrich, St. Louis, MO) in drinking water (1 mg/ml) during infection as described[26]. Control mice similarly received an equal volume of PBS. 1-MT solutions were filter-sterilized and provided to cohorts of mice beginning the day of infection and continuing until death. The corneas were monitored daily using a slit lamp equipped with a digital camera, recorded disease score for each mouse after infection (1, 3, and 5 days), and assessed according to a 12-point scoring system[27]. In brief, the disease was scored according to area of corneal opacity, density of corneal opacity, and surface regularity, each of which was given a grade of 0–4, with the highest score for uniform opacity in over three-quarters of the corneal area, perforation (never seen in this study), and descemetocele.

### Quantitative Real-time PCR

The corneal epithelial scrapings or cultured cells were harvested and saved at −80°C. The total RNA of isolated cells was extracted using RNAiso plus reagent (TaKaRa) and rapidly quantified using spectrophotometry. Complementary DNA was generated by reverse transcription of 2 μg of total RNA and then used in the following quantitative PCR reactions with SYBR Green using specific primers: 95°C for 30 s, followed by 40 cycles of 95°C for 5 s, 60°C for 30 s, and a final stage of 95°C for 15 s, 60°C for 30 s, and 95°C for 15 s. The oligonucleotide primers were as follows: β-actin, TAACACCCAGCACAATGAA and CTAAGTCATAGTCCGCCTAGAAGCA; IDO, AGAC TGTG TCTT GGCA AACT GGAA and TGCA TTGC CTTG AATA CAGT AGGA A; IL-1β, GCTGATGGCCCTAAACAGATGAA and TCCATGGCCACAACAACTGAC; IL-6, AAGCCAGAGCTGTGCAGATGAGTA and TGTCCTGCAGCCACTGGTTC. The gene expressions were quantified by qRT-PCR using the housekeeping gene β-actin as an internal control. Quantification was performed using the 2^−ΔΔCt^ method. Each experiment was repeated at least three separate times.

### Immunofluorescence

Tissue samples were fixed in 4% formaldehyde, embedded in paraffin, and cut into 3 μm- thick serial tissue sections. Non-specific binding was blocked using normal goat serum diluted 1:100 with PBS. Tissue proteolysis was performed by treatment with 0.1% protease (protease XIV, EC 3.4.24.31, Sigma, Vienna, Austria) in 0.05 M Tris-HCl, pH 7.6. After washing in EDTA-buffered saline (pH 7.6), sections were incubated with monoclonal mouse anti-human IDO antibodies diluted 1:200 overnight at 4°C. This was followed by FITC-conjugated affinipure goat anti-mouse secondary antibody (Millipore, Billerica, MA; 1:100, 1.5h, room temperature) without light. Isotype IgG was used as the negative controls. The fluorescent micrographs were taken with a Zeiss Axiovert microscope at 20× magnification.

### Western blots analysis

Cell culture proteins were extracted via RIPA lysis buffer plus 1mM PMSF at 4°C for 40 min. The lysate was centrifuged every 10 min, followed by centrifugation at 14,000 rpm for 15 min at 4°C. Total protein was quantified via bicinchoninic acid assay, denatured with sodium dodecyl sulfate–polyacrylamide gel electrophoresis (SDS–PAGE) sample loading buffer at 95°C for 5 min. Proteins (60 μg/well) were separated by 12% SDS–PAGE in Tris/glycine/SDS buffer and electroblotted onto polyvinylidene fluoride membranes (Millipore, Billerica, MA, USA). After blocking by 1% BSA for 1 h, membranes were incubated with 1:500 diluted Mouse anti-IDO and mouse anti-β-Tubulin at 4°C overnight and then incubated with secondary antibody for 1 h. All blots were detected with BeyoECL Plus (Beyotime, Shanghai, China). Band intensity was measured by Quantity One Software (Bio-Rad, CA, USA).

### Statistical analysis

The results were represented as mean ± standard deviation. One-way analysis of variance followed by Student-Newman-Keuls test was performed using GraphPad 5.0 software. For the analysis of difference in clinical score between two groups at each time in the 1-MT and PBS treated mice, Mann-Whitney *U* test was performed. *P* < 0.05 was considered to be significant.

## Results

### IDO expression increased in human corneal epithelium with *A*. *fumigatus* infection

**Fig 1:**
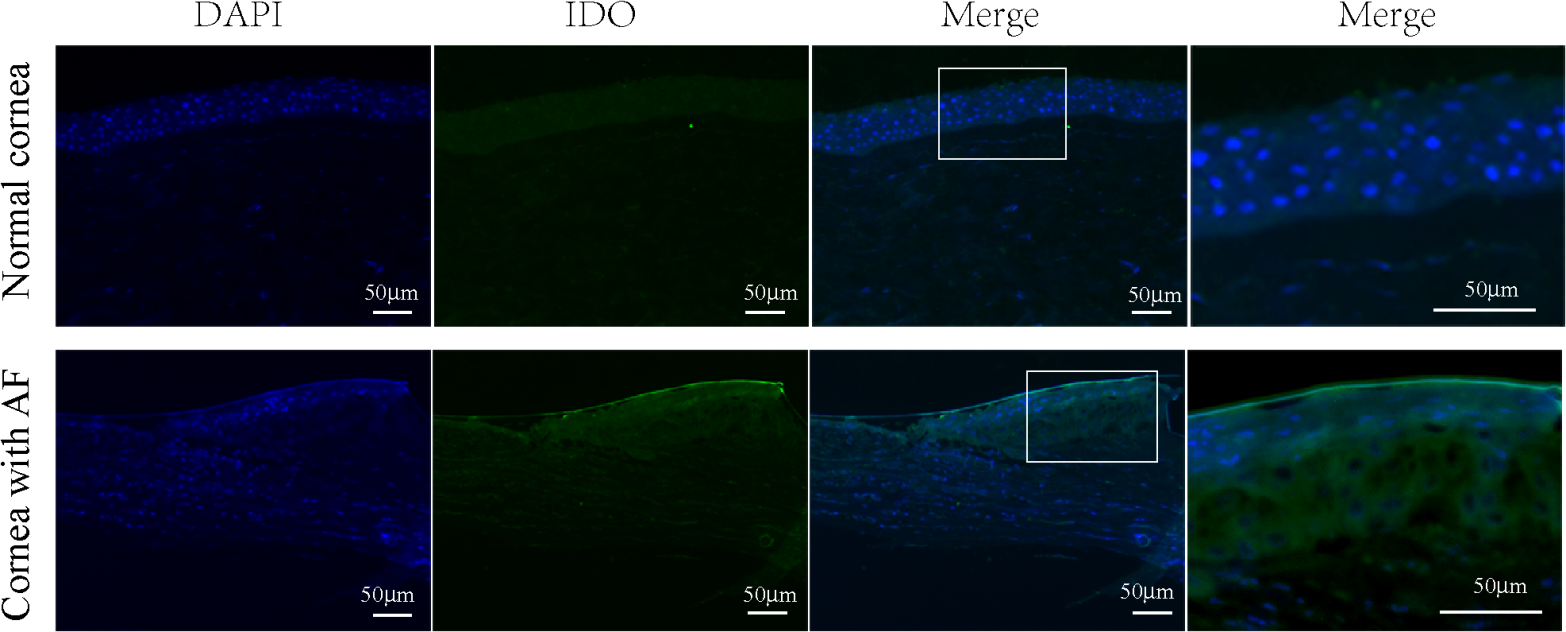
Immunofluorescence localization of IDO in normal and infected human corneas. Serial sections of normal and *A*. *fumigatus*-infected corneas were immunostained using IDO antibody and FITC-conjugated anti-mouse second antibody (for IDO, green), followed by counterstaining with DAPI (blue). Note that in normal cornea arrayed in the first row, no immunoreactivity was detected in corneal epithelium, while strong green fluorescence in *A*. *fumigatus*-infected corneal epithelium by immunofluorescent assays. Enlarged images clearly displayed IDO staining was primarily localized in the cytoplasm of corneal epithelial cells in the infected cornea. Scale bar in immunofluorescence images: 50μm.

By using immunofluorescence staining, the localization and expression of IDO were examined in corneal tissues of patients with *A*. *fumigatus* keratitis. As shown in S1B Fig, cornea in patients with *A*. *fumigatus* keratitis had dense inflammatory cell infiltration, extensive stromal destruction, and ulceration by H&E staining. IDO-positive cells in infected corneal tissues were detected with strong green fluorescence. No immunoreactivity of IDO was detected in epithelial cells of healthy corneal tissue. Enlarged images clearly displayed the IDO staining localized in the cytoplasm of the infected corneal epithelial cells (Fig 1). Primary antibody was replaced with species-specific IgG in control sections, which were negative for both groups (S1A Fig).

**Fig 2:**
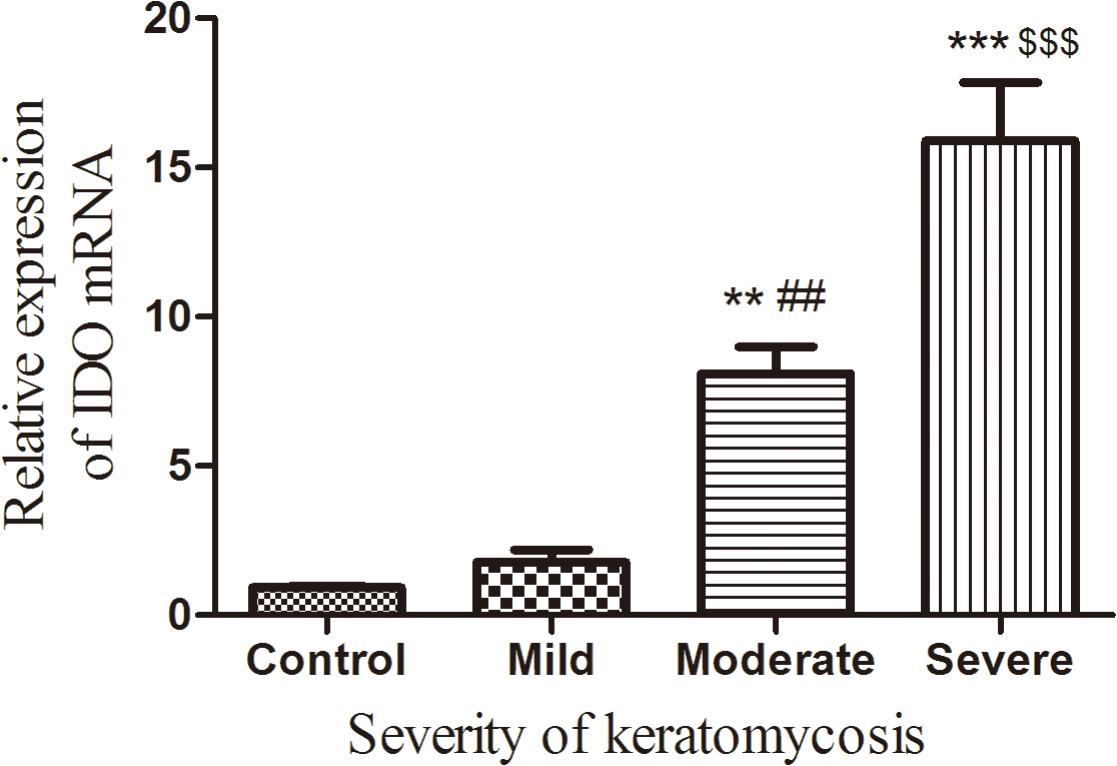
Expression of IDO mRNA in human corneal epithelium with fungal infection. qRT-PCR was performed to identify relative expression of IDO mRNA in corneal epithelium. Normal corneal tissue remainings after corneal transplantation were considered controls. Corneas with fungal infections were classified into three grades (mild, moderate and severe) according to keratomycosis severity. Fungal infection increased IDO mRNA expression in the human corneal epithelium and the expression correlated with keratomycosis severity. The data were represented as the mean ± standard deviation of four independent experiments. (**p < 0.01, ***p < 0.001 compared with control; ^##^ *p* < 0.01, compared with mild grade group; ^$ $ $^ *p* < 0.001 compared with moderate grade group).

To determine whether IDO involved in the pathogenesis of fungal keratitis, we further investigated IDO expression in 31 cases of *A*. *fumigatus* keratitis versus 6 normal human corneas. The results (Fig 2) showed that, compared with normal cornea, IDO mRNA expression was significantly increased in human corneal epithelium with fungal infection. Moreover, the expressions of IDO correlated with the severity of keratomycosis. Transcript level measured by qRT-PCR was consistent with IDO protein expression determined by immunofluorescent staining.

### IDO expression increased in HCECs incubated with *A*. *fumigatus*

**Fig 3:**
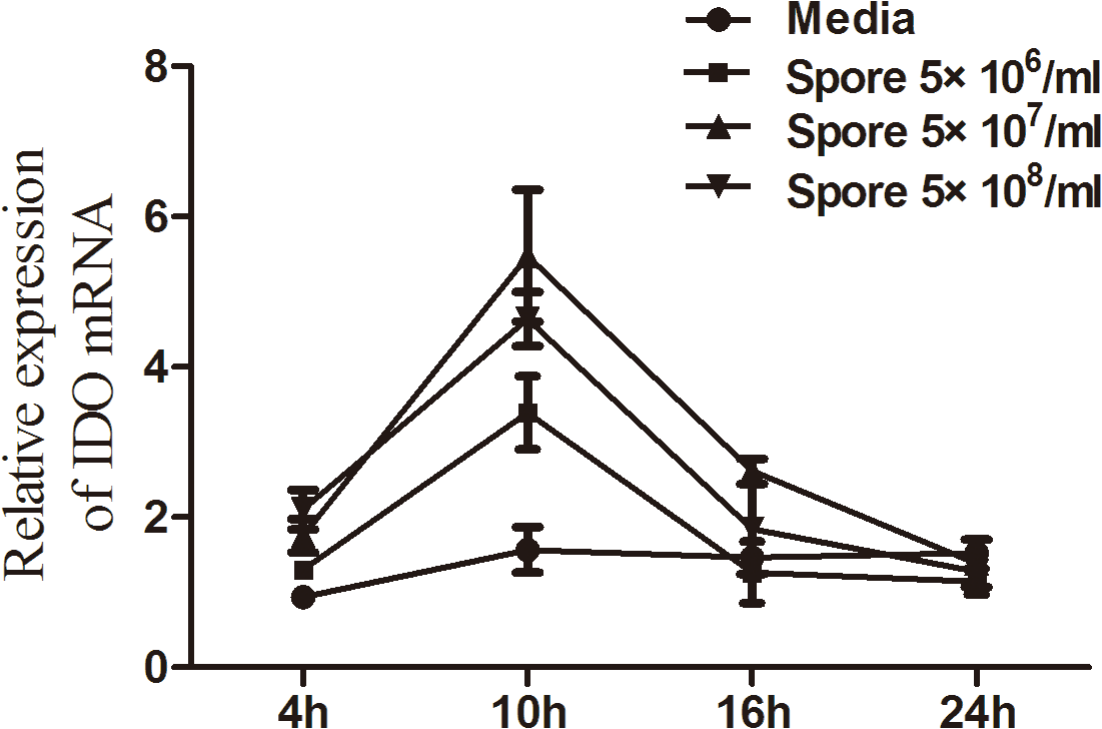
IDO expression in cultured telomerase-immortalized HCECs infected with *A*. *fumigatus*. Relative expression of IDO mRNA was detected by qRT-PCR 4, 10, 16 and 24 h after incubation with *A*. *fumigatus* spores at three different concentrations (5 × 10^6^ /ml, 5 × 10^7^ /ml and 5 × 10^8^ /ml). IDO expression increased by 4- to 5-fold with a peak level at 10 h after exposure to inactivated *A*. *fumigatus* spores (5 × 10^7^ /ml).

To explore the effect of *A*. *fumigatus* infection on IDO expressions in HCECs, we treated the cells with inactivated *A*. *fumigatus* spores and detected expressions of IDO by qRT-PCR. In order to select the optimal concentration, we stimulated HCECs with three different concentrations (5 × 10^6^ /ml, 5 × 10^7^ /ml and 5 × 10^8^ /ml) in 12-well plates. Under the stimulation of inactivated *A*. *fumigatus* spores with 5 × 10^7^/ml, IDO expressions in HCECs reached the peak at 10 h. As shown in Fig 3, the IDO mRNA was expressed in untreated primary HCECs, up-regulated at 4 h, and reached the peak level (4 to 5 fold increase from the baseline) at 10 h after exposure to inactivated *A*. *fumigatus* spores (5 × 10^7^ /ml) in HCECs. However, there was no further increase of IDO mRNA expression at 16 h.

### IDO blockade enhanced inflammatory cytokine production in HCECs and cornea of mice infected with *A*. *fumigatus*

**Fig 4:**
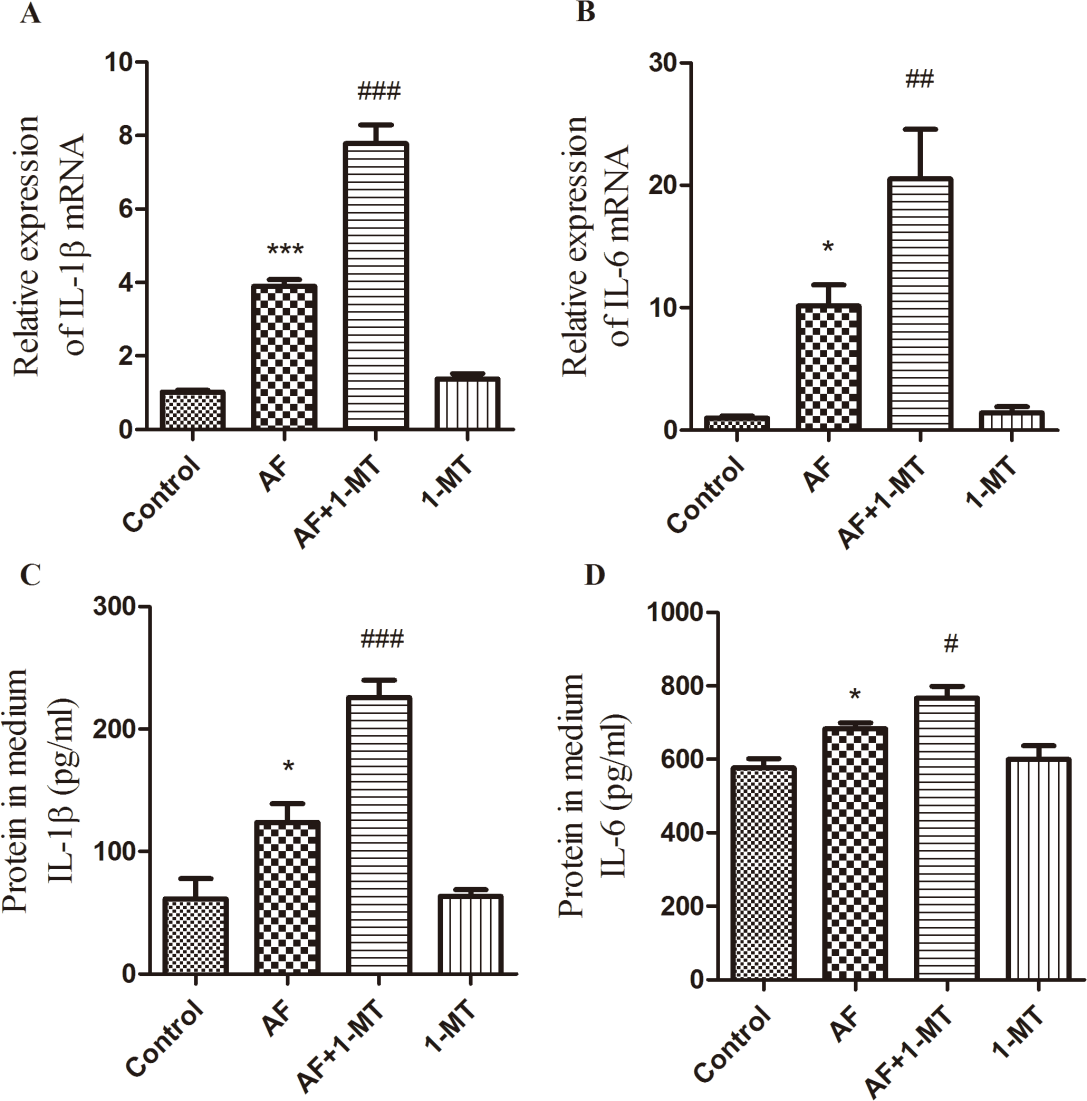
IL-1β and IL-6 expressions after blocking of IDO in cultured telomerase-immortalized HCECs infected with *A*. *fumigatus*. qRT-PCR and ELISA data demonstrated that incubation with *A*. *fumigatus* led to up-regulation of IL-1β (A, C) and IL-6 (B, D) compared with controls. However, 1-MT-induced blockade of IDO further significantly enhanced the expressions of the above inflammatory cytokines at 10 h post-infection. The data were represented as mean ± standard deviation of six independent experiments. (*p < 0.05, ***p < 0.001 compared with control; ^#^ *p* < 0.05, ^##^ *p* < 0.01, ^###^ *p* < 0.001 compared with *A*. *fumigatus* treatment group).

In order to analyze the effect of IDO on the inflammatory responses in HCECs, we examined whether 1-MT, an inhibitor of IDO, affected cytokines production in HCECs exposed to inactivated *A*. *fumigatus* spores. Cells cultured in 12- or 6-well flat bottom plates were pre-treated with 1 ng/ml 1-MT for 10 h before incubation with *A*. *fumigatus* for 10 h. Fig 4 showed that the expressions of IL-1β and IL-6 increased at 10 h post-infection with *A*. *fumigatus* in HCECs compared with the control group. Blockage of IDO by 1-MT further increased the production of these cytokines compared with the production in the *A*. *fumigatus* infection group (Fig 4A and 4B). Meanwhile, the concentration of IL-1β and IL-6 in the supernatant was also up-regulated, which was consistent with the change of IDO mRNA levels (Fig 4C and 4D).

**Fig 5:**
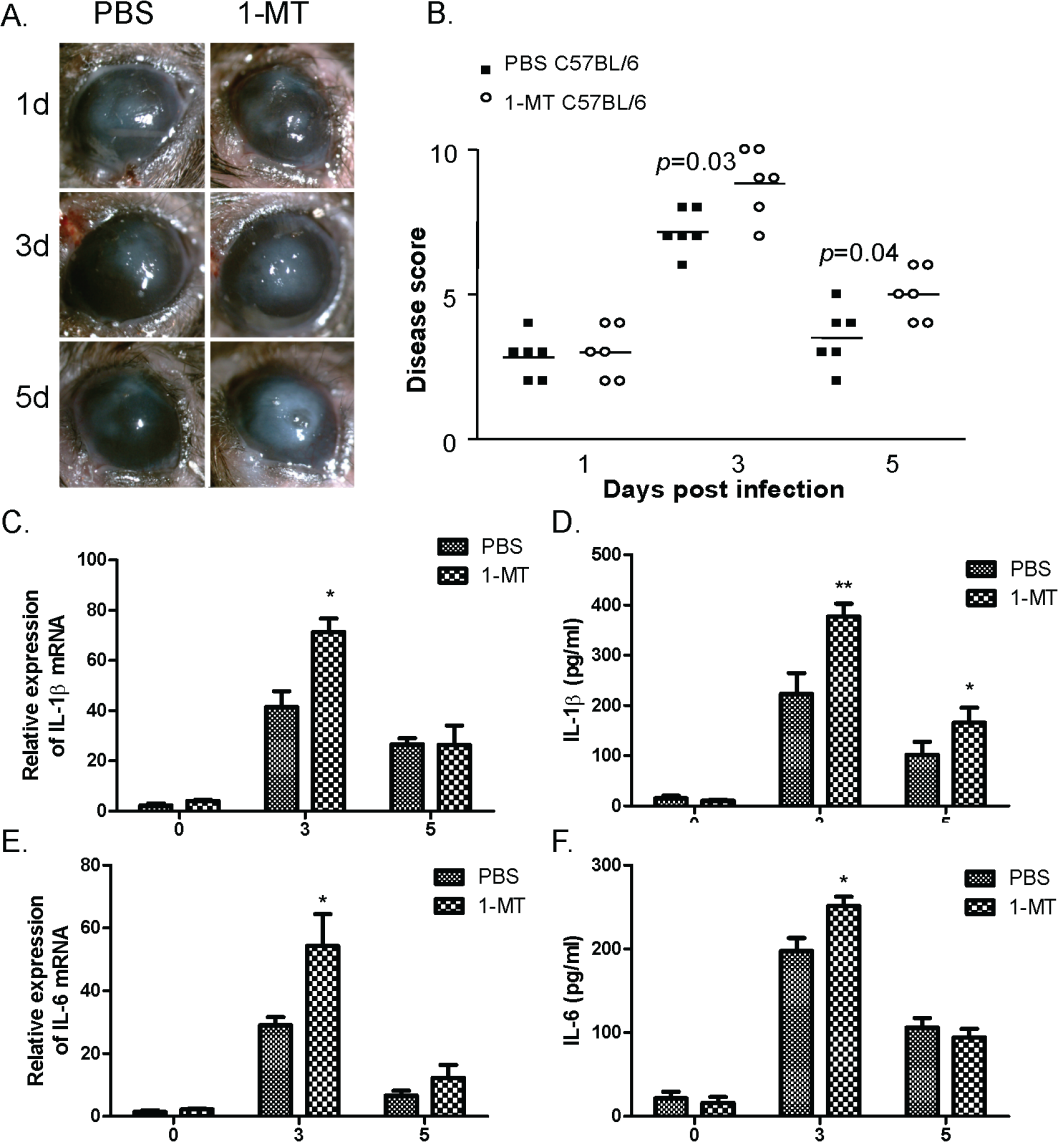
1-MT treatment enhanced inflammatory cytokine production in corneas of mice infected with *A*. *fumigatus*. C57BL/6 mice were inoculated with 2 μl of 1 × 10^5^/μl spores. (A) The corneas were monitored and taken photograps under a slit lamp, and (B) evaluated with the scoring system. Disease score is shown as mean ± standard deviation. After 1-MT treatment, the IL-1β mRNA and protein levels were significantly increased (C, D) at 3 days after infection in 1-MT treated mice compared with PBS-treated mice. In addition, both IL-6 mRNA (E) and IL-6 protein (F) were significantly increased at 3 days after infection. In addition, the IL-1β proteins were increased at 5 days after infection in 1-MT treated mice. (*p < 0.05, **p < 0.001 compared with PBS treated mice).

In HCECs incubated with *A*. *fumigatus*, inhibition of IDO could up-regulate the expressions of inflammatory cytokine IL-1β and IL-6, which prompted us to further evaluate the effect of IDO in mice cornea infected by *A*. *fumigatus*. Treatment with 1-MT increased disease severity at 3d and 5d, as indicated by disease score (Fig 5B) and slit lamp photography (Fig 5A) compared with PBS-treated mice. We further examined effects of 1-MT on inflammatory cytokine expressions in corneas infected with *A*. *fumigatus*. After treatment with 1-MT, corneal IL-1β mRNA were significantly increased (Fig 5C) at 3 days after infection compared with PBS-treated mice. Treatment with 1-MT also significantly increased IL-6 mRNA in the corneas infected with *A*. *fumigatus* compared with PBS-treated mice (Fig 5E). The expressions of IL-1β and IL-6 mRNA were down-regulated at 5 days after infection when comparing with *A*. *fumigatus* infection at 3 days in the 1-MT treatment group as well as in the PBS treatment group. Protein expressions of IL-1β and IL-6 were further tested in the corneas infected with *A*. *fumigatus* by ELISA. As shown in Fig 5D, expressions of IL-1β protein in the 1-MT treated mice were significantly increased at 3 days and 5 days after infection compared with PBS-treated mice, although partly restored at 5 days after infection. Treatment with 1-MT also significantly increased IL-6 protein in the corneas infected with *A*. *fumigatus a*t 3 days. However, no significant differences were detected in the levels of IL-6 after 1-MT treatment at 5 days (Fig 5F).

### Effects of Dectin-1 on IDO expressions in HCECs stimulated with *A*. *fumigatus* spores

**Fig 6:**
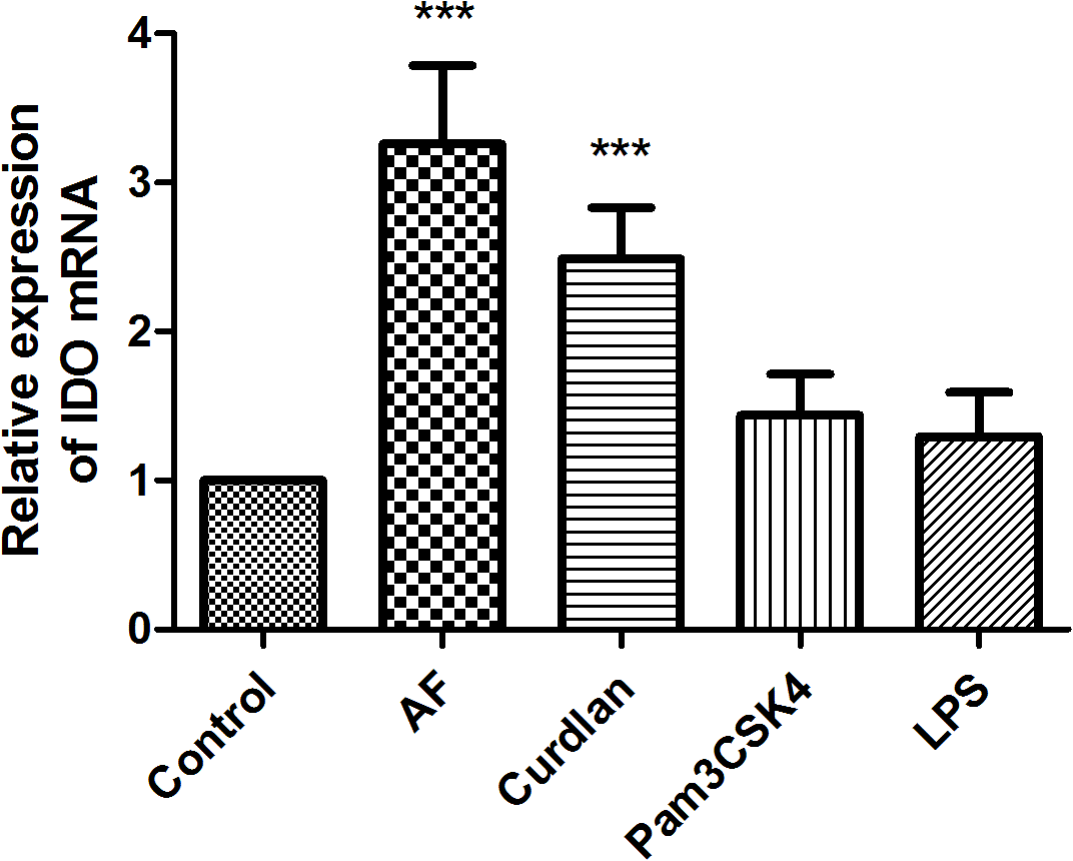
Induction of IDO by microbial ligands in HCECs. Levels of IDO mRNA at 10 h after stimulation by 200 μg/ml curdlan or 10μg/ml of Pam3CSK4, LPS were evaluated by qRT-PCR. Data were represented as mean ± standard deviation of three independent experiments. (*p< 0.05, **p < 0.01).

HCECs were incubated by a variety of extracted or synthetic microbial components, representing the ligands to Dectin-1, TLR2, TLR4 respectively for 10 h. The mRNA expression of IDO in untreated HCECs was at a relatively low level, but was largely induced (about 2- to 4-fold) after exposure to *A*. *fumigatus* and curdlan (200 μg/ml) which is the ligands to Dectin-1. The cell viability was also assessed by MTT assay after exposure to curdlan at 5, 10, 50, 100, 150, 200, 250, 300 and 500 μg/ml for 24 hours. There was no difference of cell viability when treated with curdlan between the 5–250 μg/ml, However, a signifcant reduction of cell viability was observed when treated with above 300 μg/ml curdlan (S2 Fig). IDO expression was not significantly induced by other microbial components, such as the TLR2 ligand Pam3csk4 (10 μg/ml), TLR4 ligands LPS (10 μg/ml) (Fig 6).

**Fig 7:**
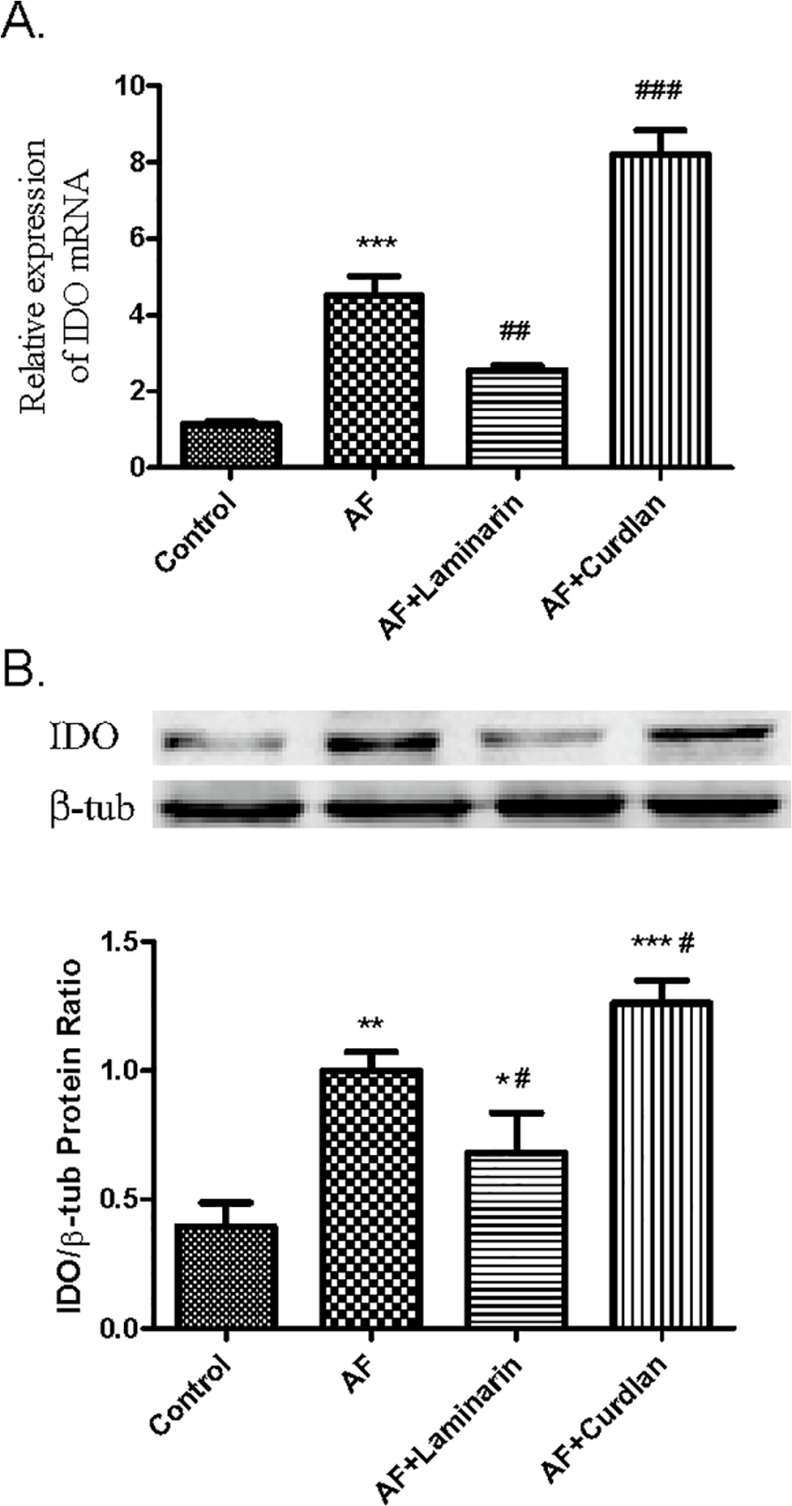
The effect of Dectin-1 on IDO expressions in cultured HCECs infected with *A*. *fumigatus*. The HCECs were pre-treated with curdlan or laminarin, and were exposed to inactivated *A*. *fumigatus* spores for 10 h. The cultures were subjected to qRT-PCR to measure IDO mRNA expressions (A). The cultures treated for 24 h were used to evaluate IDO protein expressions in HCECs by western blot (B). The data are represented as mean ± standard deviation of four independent experiments. (*p < 0.05, **p < 0.01).

To assess the contribution of Dectin-1 signaling in IDO production, we further tested effects of Dectin-1 on IDO expressions in HCECs. The cells were pre-treated with Dectin-1 ligand (curdlan, 200 μg/ml) or specific inhibitor of Dectin-1 (laminarin, 0.3 mg/ml) for 10 h and then exposed to *A*. *fumigatus* spores (5 × 10^7^/ml). As shown in Fig 7A, the mRNA expression of IDO was low in normal HCECs, however increased significantly after *A*. *fumigatus* stimulation for 10 h. The specific inhibitor laminarin at a dosage of 0.3 mg/ml (dose response studies with laminarin please see the S3 Fig) markedly reduced the stimulatory effect of *A*. *fumigatus* on IDO. Curdlan, the Dectin-1 ligand, significantly enhanced *A*. *fumigatus*-induced up-regulation of IDO. Further studies by western blot also showed that IDO expression was up-regulated by *A*. *fumigatus* stimulation for 24 h. This effect was enhanced by pre-treatment with curdlan while partly inhibited by laminarin (Fig 7B).

### Effects of 1-MT on cytokines production in HCECs stimulated with curdlan

**Fig 8:**
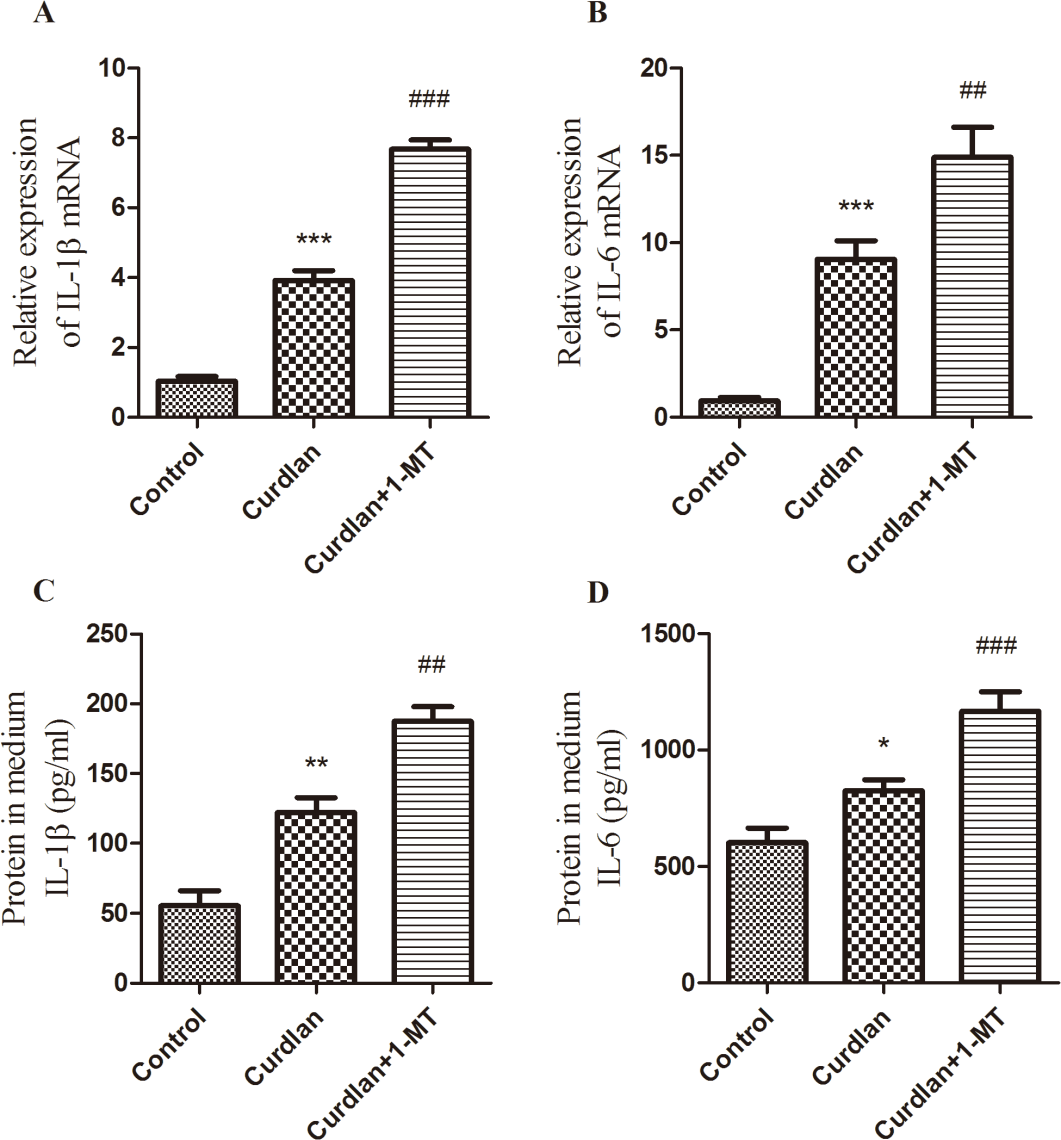
The effects of 1-MT on curdlan mediated IL-1β and IL-6 expressions in HCECs. The HCECs were pre-treated by curdlan and/or 1-MT. The cultures treated for 10 h were subjected to qRT-PCR for mRNA measurement (A, B), the cultures treated for 24 h were used to evaluate protein in the medium of 6-well by ELISA (C, D). The data were represented as mean ± standard deviation of six independent experiments. (*p < 0.05, **p < 0.01, ***p < 0.001 compared with control; ^##^ *p* < 0.01, ^###^ *p* < 0.001 compared with curdlan treatment group).

To test the role of IDO in Dectin-1 mediated inflammatory responses, the mRNA expressions of inflammatory cytokines IL-1β and IL-6 in THCEs were analyzed by qRT-PCR after curdlan treatment for 10 h. IL-1β and IL-6 expressions were significantly up-regulated by curdlan treatment compared with that of control. Blockage of IDO by 1-MT further increased the production of these cytokines compared with that of the curdlan treatment group (Fig 8A and 8B). Meanwhile, the concentration of IL-1β and IL-6 in the supernatant was measured by using ELISA. IL-1β and IL-6 production were also up-regulated in consistent with the manner of IDO mRNA levels after inhibiting the activity of IDO by 1-MT in HCECs (Fig 8C and 8D).

## Discussion

Filamentous molds, which are the major worldwide cause of fungal keratitis, are ubiquitous in the environment, and spores are present in the air we breathe[28]. Innate immune responses combine with adaptive immunity to generate the most effective form of anti-*Aspergillus* immune resistance. Although some degree of inflammation is required for protection, progressive inflammation may worsen disease and ultimately prevent pathogen eradication[29].

It had reported that the IDO enzyme had a complex role in immuno-regulation in infection[30]. IDO catalyzes the first and limiting step in the kynurenine pathway of tryptophan catabolism. While initially recognized in infection due to antimicrobial activity (“tryptophan starvation” of intracellular parasites), IDO is more importantly and widely involved in immune homeostasis of the mammalian host, and may even represent an evasion mechanism for microbes that establish commensalism or chronic infection[31,32]. This study has revealed a previously unappreciated role of IDO for HCECs against aspergillosis inflammation. In clinical human corneal tissues with fungal infection, IDO was significantly detected in inflammatory cells and corneal epithelial cells within inflamed cornea tissues. Besides, the expression of IDO increased with increasing keratomycosis severity in the human corneal epithelium with fungal infection. The results verify that IDO is involved in the pathogenesis of fungal keratitis.

To explore molecular pathways leading to or diverting from pathogenic inflammation in infection, we resorted to corneal epithelial cells, which was known as the first barrier of the cornea against pathogens and could activate distinct signaling pathways. We found that the expression of IDO in HCECs was up-regulated after exposure to inactivated *A*. *fumigatus* spores. These findings further confirm the presence of IDO in human corneal epithelial cells which may take part in fungal infection.

It was reported that IDO could have opposite roles in host defense against infection. IDO could play a dominant role in directly suppressing pathogen replication (for example during toxoplasmosis or chlamydial infections)[33,34], or by limiting the spread of virus infection[35]. However, IDO could also dampen protective host immunity, thus indirectly leading to increased pathogen burdens (e.g., as occured during leishmaniasis)[33,36]. In order to explore the function of IDO in the pathogenesis of fugal keratitis, we further investigated whether 1-MT, an inhibitor of IDO, affected cytokine production in HCECs exposed to inactivated *A*. *fumigatus* spores. We observed that 1-MT significantly increased the production of IL-1β and IL-6 after *A*. *fumigatus* infection compared with the production in the *A*. *fumigatus* group. These results indicated that IDO up-regulation induced by *A*. *fumigatus* was involved in the course of infection by modulation of host proinflammatory and immune responses.

During infection, host immune cells recognize evolutionarily conserved microbial components called “microbial associated molecular patterns (MAMPs)” through germline encoded PRRs[6,37,38], activate innate defence mechanisms[6,38,39] by intracellular signaling transmission, initiate phagocytosis and inflammatory responses, and generate specific and consistent adaptive immune response to resist the fungal infection[40]. Dectin-1, as one of the important PRRs, is a type II transmembrane receptor and involved in the recognition of fungal cell wall. The carbohydrate recognition domains (CRD) selectively bind with fungal β-glucan polymers, a major component of yeast cell wall, and induce phagocytosis[39,41,42]. Previous studies showed that Dectin-1 expression was elevated in human tissue infected with *Aspergillus* or *Fusarium*, which was consistent with the results of the studies using a murine model of *A*. *fumigatus* keratitis in which Dectin-1 was found to mediate cytokine production, neutrophil recruitment, and fungal survival[7]. In order to assess the contribution of Dectin-1 signaling to IDO production, we used curdlan and laminarin to pretreat HCECs before incubation with *A*. *fumigatus* spores. Curdlan, Dectin-1 agonist with immunomodulatory properties, can activate dendritic cells to induce the expression of IDO and a distinct profile of cytokine secretion[43–45], while laminarin is commonly used as a Dectin-1 antagonist[46–48]. Interestingly, *Hiroyuki et al* have reported the similar effects of laminarin and curdlan on Dectin-1 stimulation. However, the experiments were done on the SKG mice with immunodeficiency due to a mutation of the gene ZAP-70[49]. The difference of the results might be attributed to the immune state of the mice. Further experiments should be carried out to investigate the possible mechanisms involved. Consistent with previous studies, we showed that *A*. *fumigatus* in combination with Dectin-1 ligands curdlan enhanced the expression of the IDO protein, while Dectin-1 specific inhibitor laminarin inhibited the expression of the IDO protein. These findings suggested that Dectin-1 may modify IDO expression in the corneal epithelial cells. We also showed that the inhibitor of IDO further enhanced IL-6 and IL-1β production in curdlan–stimulated corneal epithelial cells, suggesting the IDO expression is involved in Dectin-1 mediated cytokines production in the corneal epithelial cells. Taken together, the findings of our study indicate that the interaction of PRRs and *A*. *fumigatus* could be concerned with irreversible change of keratitis via cytokine expression such as IL-6 and IL-1β in concert with IDO expression.

In conclusion, our studies have revealed the contribution of HCECs to protective immunity to Aspergillus through a Dectin-1-dependent pathway converging on IDO. We provide the direct evidence that HCECs may be able to induce IDO for the proper control of the infection and the associated inflammatory response.

## Supporting Information

S1 Fig A. Isotype control of immunofluorescence staining of IDO in normal and infected human corneas. B. H&E staining of human corneas of *A*. *fumigatus* keratitis. Arrow depicts inflammatory cell infiltration, extensive stromal destruction, and ulceration.
(TIF)

S2 Fig Cell viability assay in HCECs after exposure to curdlan. (TIF)

S3 Fig Dose-response studies with laminarin in HCECs. (TIF)
