# Supplementary figures and images for: Indoleamine 2,3-Dioxygenase Is Involved in the Inflammation Response of Corneal Epithelial Cells to Aspergillus fumigatus Infections
Source: PLoS One. 2015 Sep 11;10(9):e0137423. doi: 10.1371/journal.pone.0137423 (PMC4567309; doi:10.1371/journal.pone.0137423)

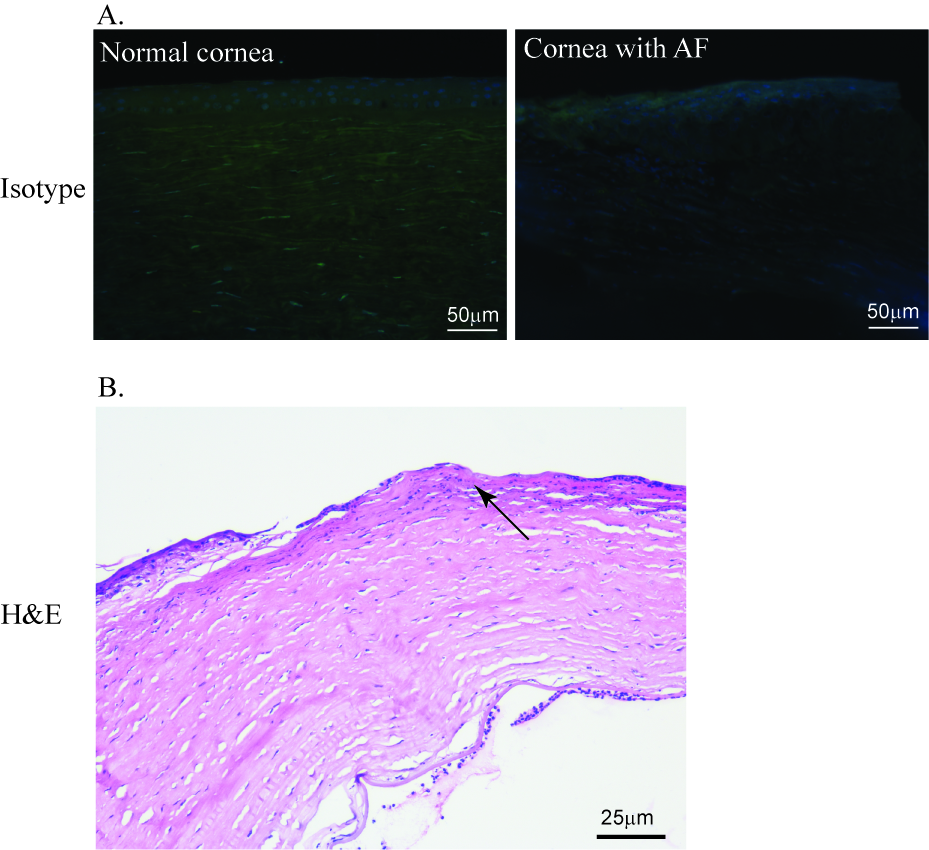

Supplement: S1 Fig — Arrow depicts inflammatory cell infiltration, extensive stromal destruction, and ulceration. (TIF) [file pone.0137423.s001.tif]

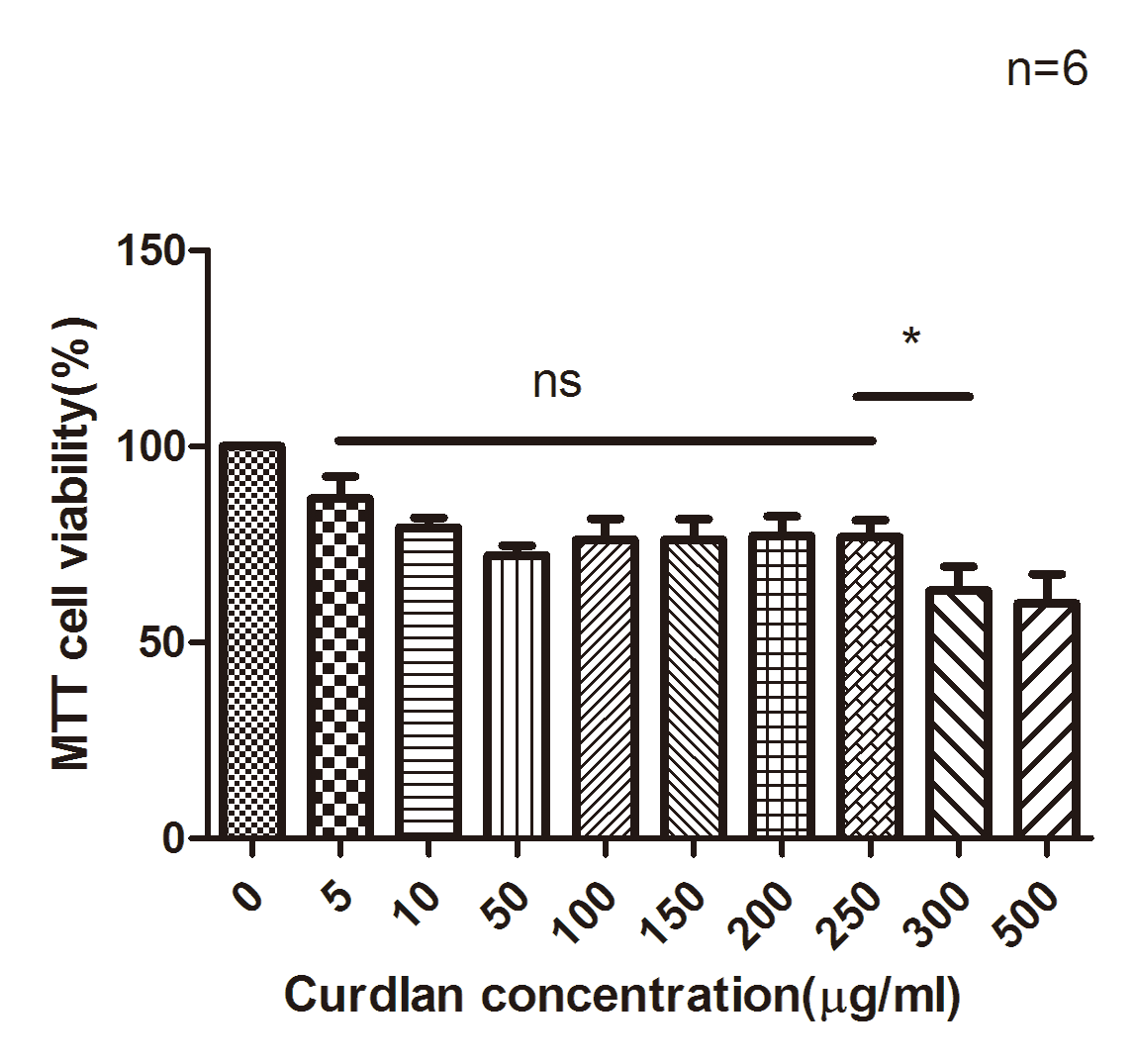

Supplement: S2 Fig — (TIF) [file pone.0137423.s002.tif]

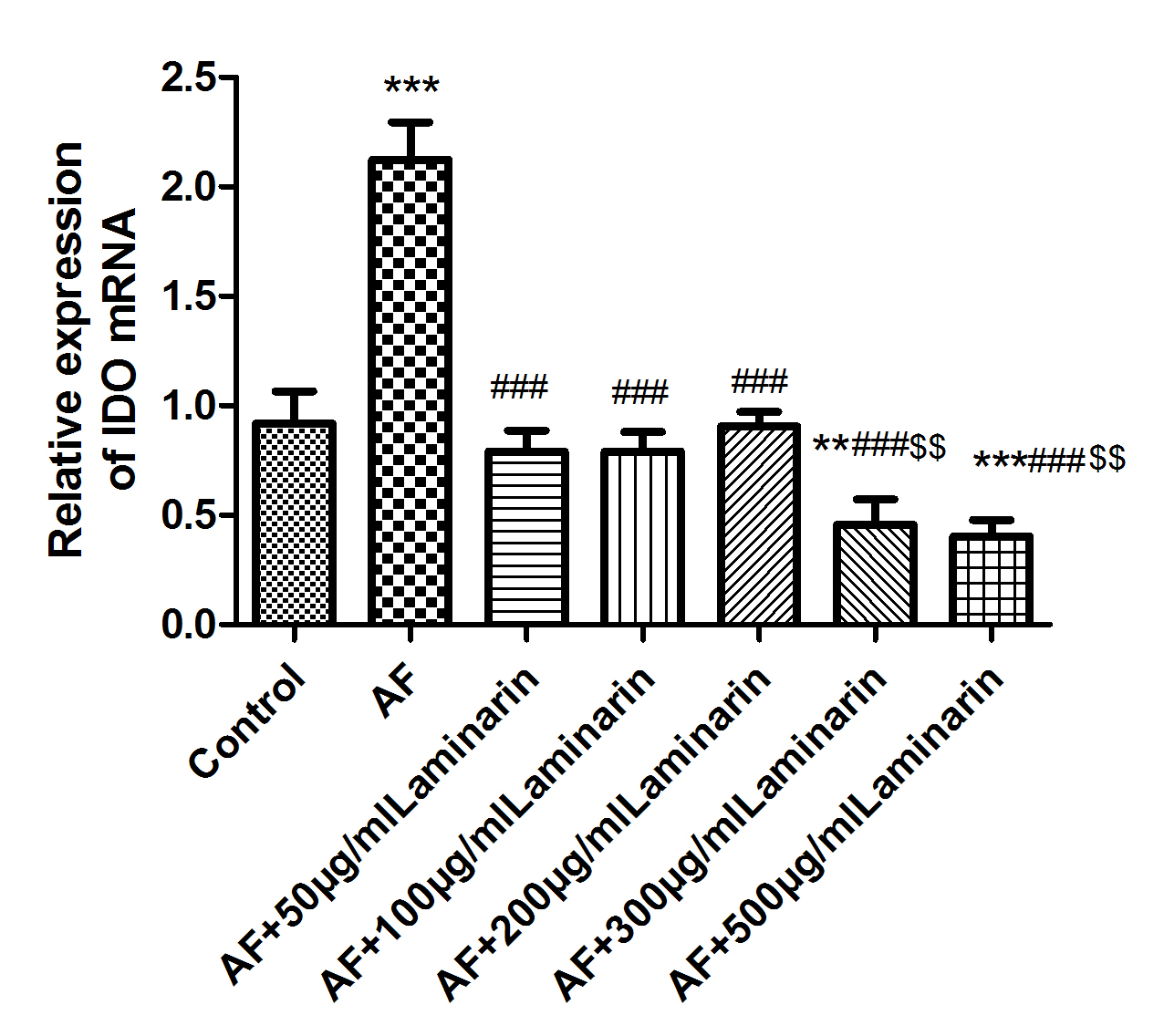

Supplement: S3 Fig — (TIF) [file pone.0137423.s003.tif]
